# Structural Insights into the Nucleotide-Binding Domains of the P_1B_-type ATPases HMA6 and HMA8 from *Arabidopsis thaliana*

**DOI:** 10.1371/journal.pone.0165666

**Published:** 2016-11-01

**Authors:** Hubert Mayerhofer, Emeline Sautron, Norbert Rolland, Patrice Catty, Daphné Seigneurin-Berny, Eva Pebay-Peyroula, Stéphanie Ravaud

**Affiliations:** 1 Institut de Biologie Structurale (IBS), Université Grenoble Alpes, CEA, CNRS, Grenoble, France; 2 Laboratoire de Physiologie Cellulaire et Végétale (LPCV), Institut de Biosciences et Biotechnologies de Grenoble (BIG), Université Grenoble Alpes, CEA, CNRS, INRA, Grenoble, France; 3 Laboratoire de Chimie et Biologie des Métaux (LCBM), BIG, Université Grenoble Alpes, CEA, CNRS, INRA, Grenoble, France; University of Saskatchewan, CANADA

## Abstract

Copper is a crucial ion in cells, but needs to be closely controlled due to its toxic potential and ability to catalyse the formation of radicals. In chloroplasts, an important step for the proper functioning of the photosynthetic electron transfer chain is the delivery of copper to plastocyanin in the thylakoid lumen. The main route for copper transport to the thylakoid lumen is driven by two P_IB_-type ATPases, **H**eavy **M**etal **A**TPase 6 (HMA6) and HMA8, located in the inner membrane of the chloroplast envelope and in the thylakoid membrane, respectively. Here, the crystal structures of the nucleotide binding domain of HMA6 and HMA8 from *Arabidopsis thaliana* are reported at 1.5Å and 1.75Å resolution, respectively, providing the first structural information on plants Cu^+^-ATPases. The structures reveal a compact domain, with two short helices on both sides of a twisted beta-sheet. A double mutant, aiding in the crystallization, provides a new crystal contact, but also avoids an internal clash highlighting the benefits of construct modifications. Finally, the histidine in the HP motif of the isolated domains, unable to bind ATP, shows a side chain conformation distinct from nucleotide bound structures.

## Introduction

Heavy metals, with their unique chemical properties, play an essential role in cellular processes as cofactors, structural stabilizers or redox partners and plants have evolved to maintain physiological concentrations of crucial metal ions. Their concentration and distribution needs to be tightly regulated as a large fraction of the proteome depends on them, but also to avoid their toxic effects when present in excess. Therefore, a number of cellular mechanisms exist ensuring their proper handling ranging from uptake and trafficking to storage and sequestration. As metal ions cannot diffuse freely across membranes, transporters play an important role in their targeting to different subcellular compartments.

P-type ATPases are transmembrane proteins that play a key role in the uphill transport of a wide range of cations across membranes using the energy provided by the hydrolysis of ATP, being crucial for the ion homeostasis in nearly all organisms and for heavy metal detoxification [1]. The catalytic cycle follows the classical Post-Albers model [2] and consists of four main steps with an alternating opening of the channel on the different sites of the membrane and the transport of the cation through extensive conformational changes, coupling ATP hydrolysis and cation transport. It includes the binding of ATP by the nucleotide (N) binding domain and its proper positioning for the transient phosphorylation of an aspartate within the invariant cytoplasmic DKTGT motif, part of the Phosphorylation (P) domain, and the subsequent dephosphorylation assisted by the Actuator (A) domain [3]. Conformational changes not only occur at the catalytic part of the transporter but also at its membrane domain affecting the access and the affinity of the transport site and leading to the motion of an ion. The P-type ATPase family can be divided into 5 groups [4]. The P_1B_ subfamily, one of the largest, was shown to transfer transition metals (Cu^+^, Ag^+^, Cu^2+^, Zn^2+^…) and can be further divided into five subgroups with varying specificities. They consist of eight transmembrane (TM) helices, three cytoplasmic domains (A, N and P domain) and of a varying number of metal-binding domains at one of the termini. Characteristic features of the subfamily comprise a C/SPC motif found in TM6, cytosolic domains that are considered to be smaller than for other subfamilies [5], a particular membrane topology and conserved residues important for ATP binding, including a HP motif [6], different from the other families, while residues conserved elsewhere are not preserved.

Copper (Cu) is redox-active and plays an important role in electron-transfer reactions including the photosynthetic electron transport. It is also involved in the formation of reactive oxygen radicals and its free form is therefore tightly regulated, with virtually no free copper available in a cell. In plants, about half copper is present in the chloroplast either bound to the Cu/Zn superoxide dismutase (in the stroma) or to the plastocyanin located in the thylakoid lumen, with both proteins acting also as a copper sink in cases of abundant supply [7]. Plastocyanin is a small soluble protein, transferring electrons between cytochrome *f* of the cytochrome *b*_*6*_*/f* complex and the reaction centre of photosystem I. Two isoforms of plastocyanin are found in *Arabidopsis thaliana* [8]. Eight members of the heavy metal P_1B_-type ATPases subclass are present in *A*. *thaliana*, three of them being located into the chloroplast [9]. HMA1 and HMA6 are found in the inner membrane of the chloroplast envelope and HMA8 in the thylakoid membrane. HMA1 belongs to subgroup P_1B-4_, while HMA6 and HMA8 belong to subgroup P_1B-1_. HMA6 represents the main route for copper into the chloroplast, also supplying the Cu/Zn superoxide dismutase [10,11], while HMA1 represents a second route for copper, crucial under high light [12]. HMA8 supplies plastocyanin in the thylakoid lumen with copper, which also controls the stability of HMA8, but not of HMA6 [13,14]. HMA6 and HMA8 are both needed for an efficient photosynthetic electron transport [14]. However, they exhibit differing catalytic properties, HMA8 having a higher affinity for Cu and lower dephosphorylation kinetics than HMA6 [15].

Structures of different members of the P-type ATPase family exhibit the same overall fold, but differences exist between the subgroups [3] and, up to date, only few structures of the P_1B_ subfamily are available [16–18]. Structural information for plant proteins from the subfamily is basically missing. Here, we present two structures of the nucleotide binding domain of HMA6 and HMA8, the former at the highest resolution, obtained for any P-type ATPase structure. They exhibit a very compact fold forming the minimum ATP binding unit. The conserved residues specific to the subfamily are spatially well aligned to other structures, yet a shift in the position of the conserved His in the HP motif could explain the inability of the isolated domain to bind ATP and can be a way of regulating the nucleotide binding. The surfaces surrounding the entrance of the nucleotide-binding cavity exhibit notable differences in their charge distribution, which might affect the binding of ATP and subsequently the copper pumping rate.

## Materials and Methods

### Cloning and purification of HMA6^N^ and HMA8^N^

The cDNA sequences coding for the nucleotide-binding domains of HMA6 (HMA6^N^, residues 607–734) and HMA8 (HMA8^N^, residues 557–689) were subcloned from the full-length constructs. The sequences of the primers used were: HMA6-forward 5'- CAGGGCGCCAGTCACCCTGTTGTGACTGAAGTTATTATTCCTG-3’, HMA6-reverse 5'- GACCCGACGCGGTTATTCAAAACGGATGACTGCAGCAAG-3’, HMA8-forward 5'- CAGGGCGCCAGTAGACCTGTCGTCTCTGGTGTTG-3’ and HMA8-reverse 5'- GACCCGACGCGGTTAATCGGATATTGCAATAGCACCAATGATCC-3’.

The primers contain appropriate extensions for ligation independent cloning (LIC). The gene was inserted into a pETM-11/LIC vector (A. Geerlof, Helmholtz Zentrum München) via LIC cloning. The vector contains an *N*-terminal His6-tag followed by a Tobacco Etch Virus protease (TEV) cleavage site in case of HMA8^N^ or an GB1 expression and solubility enhancer [19] flanked by a *N*-terminal His6-tag and a TEV cleavage site in case of HMA6^N^, with a three amino acid linker (GAS) that remains at the *N*-terminus of both proteins after TEV cleavage. Two glutamate residues were mutated to alanine in the HMA6^N^ construct (EE709AA) using the QuikChange site-directed mutagenesis kit (Stratagene), yielding the HMA6^N^-AA construct. The final constructs were verified by DNA sequencing.

The plasmids were transformed into the *E*. *coli* Rosetta strain. A colony from freshly transformed cells was used to inoculate 5 mL lysogeny broth medium containing kanamycin (50 μg.mL^-1^) and grown overnight at 310K. The overnight HMA8^N^ culture was diluted to 1/1000^th^ into 2 L of terrific broth medium containing kanamycin. They were grown to optical densities between 0.6–0.7 at 600 nm after which the temperature was lowered to 293K. The bacteria were induced using 0.05 mM isopropyl β-D-1-thiogalactopyranoside. The overnight HMA6^N^-AA culture was harvested at 2000 *g* for 5 min followed by resuspension in M9 minimal media. This was used to inoculate 2 L of M9 media supplemented with 5 g.L^−1^ glucose, 50 μg.L^-1^ kanamycin, 1 mg.L^−1^ thiamine and 1 mg.L^−1^ biotin. At an OD_600_ of 0.5, 100 mg.L^−1^ each of phenylalanine, lysine and threonine, 50 mg.L^−1^ each of leucine, isoleucine, valine and l-SeMet were added [20]. At an OD_600_ of 0.6 the culture was induced with 0.15 mM IPTG after which the temperature was lowered to 301K. Cultivation was continued in both cases for 16 h and cells were harvested by centrifugation at 5000 rpm in a JLA-8.1000 rotor for 20 min at 277K. The cell pellets were stored at 253K. After thawing on ice, pellets were re-suspended in lysis buffer (20 mM Mops pH 7.0, 250 mM NaCl, 10% (w/v) glycerol, 20 mM imidazole, 1 mM complete protease inhibitor cocktail EDTA-free (Roche), 1 mg.mL^−1^ DNAse (NEB) and 0.1% (w/v) CHAPS (Euromedex)) in a final volume of 20 mL per 5 g of cells and lysed by pulsed sonication on ice for two times 90 sec. The lysates were centrifuged at 37,000 rpm in a Ti-45 rotor at 277K for 60 min. The supernatants were loaded onto a 5 mL HisTrap HP column (GE Healthcare), which had been equilibrated against buffer A (20 mM Mops pH 7.0, 250 mM NaCl, 5% (w/v) Glycerol). The column was washed with 5 column volumes (CV) of buffer A, followed by 5 CV of buffer A with 20% of buffer B (buffer A with 250 mM imidazole). The proteins were eluted with a gradient of 20% to 100% of buffer B in buffer A (50 mM to 250 mM imidazole) within 13 CV. Fractions containing HMA8^N^ or HMA6^N^-AA were pooled and dialysed overnight at 277K against buffer C (20 mM Mops pH 7.0, 150 mM NaCl and 6 mM β-ME) or buffer D (20 mM potassium phosphate buffer pH 7.0, 150 mM NaCl and 6 mM β-ME), respectively, in a dialysis bag (molecular-weight cut-off 5 kDa) with 1 mg TEV protease (containing a non-cleavable *N*-terminal His-tag) added per 30 mg of protein. The solution was collected the next day and passed again over the Ni-NTA column equilibrated against buffer C or D. The flow-through was collected and concentrated using a Vivaspin column (molecular-weight cutoff 5kDa). Size-exclusion chromatography (HiLoad 26/60 Superdex 75, Amersham Biosciences) was used as a final step of purification. The column was pre-equilibrated in buffer E (20 mM Mops pH 7.0, 150 mM NaCl) in case of HMA8^N^ or buffer E (20 mM potassium phosphate buffer pH 7.0, 150 mM KCl) in case of HMA6^N^-AA. The samples eluted as a single peak and peak fractions were analysed by SDS-PAGE and pooled.

### Crystallization of HMA8^N^

HMA8^N^ was concentrated to 35 mg.mL^-1^ using a Vivaspin column (molecular-weight cut-off 5kDa). Initial crystallization trials were carried out with 5 different 96 well screens (Wizard I+II (Rigaku Reagents), Crystal Screen Lite & PEG/Ion and Grid screen (both Hampton Research), JCSG and PACT (both Qiagen) screens) at the High-throughput crystallisation laboratory (HTX Lab, EMBL, PBS, Grenoble). All initial screens were performed at 293K in 96-well Greiner plates using the sitting-drop vapour-diffusion method. 100 nl protein solution was mixed with 100 nL reservoir solution and equilibrated against 50 μL reservoir solution.

Crystallization trials with His tag-free HMA8^N^ resulted in a number of hits. Initial lead conditions were optimized through the use of custom made screens. The best crystals of HMA8^N^ were observed at 293K by mixing 1 μL of protein with 1 μL of 0.1 M sodium acetate trihydrate pH 4.4 and 0.6 M sodium formate. Co-crystallization and soaking trials using 5–10 mM ATP were also performed in the same crystallization conditions.

### Crystallization of HMA6^N^-AA

HMA6^N^-AA was concentrated to 10 mg.mL^-1^ using a Vivaspin column (molecular-weight cut-off 5 kDa). Initial crystallization trials were carried out with 4 different 96 well screens (Wizard I+II (Rigaku Reagents), JCSG and PEGs (both Qiagen)) screens at the High-throughput crystallisation laboratory (HTX Lab, EMBL, PBS, Grenoble) [21]. All initial screens were performed at 293K in 96-well Greiner plates using the sitting-drop vapour-diffusion method. 100 nL protein solution was mixed with 100 nL reservoir solution and equilibrated against 50 μL reservoir solution.

Crystallization trials with His tag-free HMA6^N^-AA resulted in one hit. Initial lead conditions were optimized through the use of custom made screens, which involved microseeding, using seeds obtained from the initial hit, and varying the protein concentration. The best crystals of HMA6^N^-AA were observed at 293K by mixing 1 μL of protein at 4 mg.mL^-1^ with 1 μl of 0.1 M sodium acetate trihydrate pH 4.4 and 0.6 M sodium formate. The plate was incubated at 293K for 4 h after which the wells were reopened and, using a cat whisker, microseeds were added. Crystals appeared after 2 days and reached their final size after about 1 week.

### Data collection and processing

Prior to data collection, crystals of HMA8^N^ were cryoprotected with Paratone-N, while crystals of HMA6^N^-AA were cryoprotected by adding ethylene glycol to the reservoir solution to a final concentration of 27% (w/v). The crystals were then flash-cooled to 77K in liquid nitrogen. A complete X-ray diffraction data set was collected on beamline BM14 at the European Synchrotron Radiation Facility (ESRF, Grenoble, France) using a MARCCD 225 detector in case of HMA8^N^. A total of 150 frames were collected with a rotation range of 1° and a detector distance of 187.25 mm. For HMA6^N^-AA complete X-ray diffraction data sets corresponding to the peak and inflection point were collected on beamline ID23-1 (ESRF, Grenoble, France) using a PILATUS 6M-F detector. A total of 1210 frames were collected with a rotation range of 0.1° and a detector distance of 271.22 mm. The data were indexed and integrated using *XDS* [22] and scaled with *Aimless* [23].

### Structure solution and refinement

The structure of HMA8^N^ was solved by molecular replacement using the N-domain of the CopA structure from *A*. *fulgidus* as a search model (pdb file 3A1C) in PHASER [24]. The model was manually modified in COOT [25] and refined using Phenix 1.10_2155 [26] by TLS refinement with 22 TLS groups that had been determined by the program [27]. Multiwavelength anomalous dispersion phasing using the SeMet-substituted crystal solved the structure of HMA6^N^-AA. SeMet sites were found with SHELXD and density modifications were performed with the program SHELX E [28]. Using the traceable map automated model building was carried out using buccaneer [29], which traced 80% of the structure. Further building was performed cycling between manual modelling in COOT [25] and refinement in Phenix 1.10_2155 [26], using only the dataset collected at the peak wavelength. MolProbity [30] analysis indicated that the overall geometry of the final model of HMA8^N^ and HMA6^N^-AA ranked in the 97^th^ and 84^th^ percentile, respectively, where the 100^th^ percentile is best among structures of comparable resolution. All structure-related figures were generated with PYMOL. Data collection and refinement statistics for HMA6^N^-AA and HMA8^N^ are shown in Tables 1 and 2, respectively. The coordinates are deposited in the PDB under the accession codes 5LBD and 5LBK corresponding to HMA6^N^ and HMA8^N^, respectively.

**Table 1:** Data collection and refinement statistics of HMA6^N^-AA.

Data collection	Peak	Inflection	Remote
Diffraction source	ID23-1	ID23-1	ID23-1
Wavelength (Å)	0.97924	0.97947	0.97692
Temperature (K)	100	100	100
Crystal-detector distance (mm)	271.40	271.22	272.21
Rotation range per image (°)	0.1	0.1	0.1
Total rotation range (°)	121	121	121
Space group	P 2 2_1_ 2_1_	P 2 2_1_ 2_1_	P 2 2_1_ 2_1_
*a*, *b*, *c*	38.77 65.15 85.6	38.79 65.23 85.64	38.84 65.30 85.68
α, β, γ	90 90 90	90 90 90	90 90 90
Mosaicity (°)	0.062	0.068	0.083
Resolution range	50–1.50 (1.53–1.50)	50–1.50 (1.53–1.50)	50–1.50 (1.53–1.50)
Total No. of reflections	155329 (7508)	155325 (7597)	155309 (7467)
No. of unique reflections	35341 (1689)	35433 (1707)	35474 (1689)
Completeness (%)	99.5 (99.0)	99.6 (99.4)	99.5 (97.9)
Redundancy	4.4 (4.4)	4.4 (4.5)	4.4 (4.4)
<I/σ(I)>	13.4 (1.4)	13.6 (1.5)	15.7 (1.1)
**Refinement statistics**			
R factor	18.54 (32.23)		
Rfree	20.72 (34.6)		
No. of non-H atoms			
Protein	1761		
Water	149		
Mean B factors (Å^2^)			
Protein	30.86		
Ligands	44.8		
Solvent	38.4		
R.m.s. deviation from ideal			
Bond length (Å)	0.01		
Bond angles (°)	0.922		
Ramachandran plot (%)			
Residues in favoured region	97.76		
Residues in allowed region	2.24		
Outliers	0		
Molprobity Score	1.52		
PDB code	5LBD		

**Table 2:** Data collection and refinement statistics of HMA8^N^.

**Data collection**	
Diffraction source	BM14 (ESRF)
Wavelength (Å)	0.953
Temperature (K)	100
Detector	MARCCD 225
Crystal-detector distance (mm)	160.85
Rotation range per image (°)	1.0
Total rotation range (°)	150
Space group	P2_1_2_1_2_1_
*a*, *b*, *c*	44.157 48.257 108.295
α, β, γ	90, 90, 90
Mosaicity (°)	0.134
Resolution range	34.22–1.756 (1.86–1.756)
Total No. of reflections	141918 (22757)
No. of unique reflections	23737 (3786)
Completeness (%)	99.4
Redundancy	6.0 (6.0)
<*I*/σ(*I*)>	16.5 (2.64)
**Refinement statistics**	
R factor	19.81
R_free_	23.76
No. of non-H atoms	
Protein	1787
Water	125
Mean B factors (Å^2^)	
Protein	43
Water	43.6
R.m.s. deviation from ideal	
Bond length (Å)	0.013
Bond angles (°)	1.103
Ramachandran plot (%)	
Residues in favoured region	97.4
Residues in allowed region	2.6
Outliers	0
Molprobity Score	1.35
PDB code	5LBK

### Isothermal titration calorimetry

Calorimetric measurements were tested at 20°C or 25°C under continuous stirring at 286 rpm to ensure rapid mixing, using a VP-ITC instrument with a cell volume of 1.4569 ml (MicroCal, LLC). After concentration HMA6^N^ and HMA8^N^ were dialysed against the buffer used for size-exclusion chromatography with or without the addition of 1 mM MgCl_2_. The same batch of buffer was used for further dilutions of the proteins and for the dilution of the nucleotide. The protein concentrations used in the cell were between 130 μM– 250 μM for HMA6^N^ and between 18 μM– 250 μM for HMA8^N^. As binding partner ATP was tested at concentrations between 500 μM and 8.6 mM.

## Results and Discussion

### Construct optimization and structure determination

The N-domain of HMA8 (HMA8^N^) was readily expressed and purified, representing monomeric protein, which eluted as a symmetric peak with a calculated mass of 17.2 kDa. The structure of HMA8^N^ was solved at 1.75 Å by molecular replacement, using the N-domain of pdb 3A1C as a search model, with two molecules in the asymmetric unit (AU).

The initial construct (HMA6^N^) of the HMA6 N-domain was poorly expressed and required the addition of an *N*-terminal solubility enhancing tag, which lead to a 15-fold increase in expression. After cleavage of the tag, highly soluble and homogenous protein was obtained, which eluted from the size-exclusion column as a monomer, with a calculated mass of 15.4 kDa, yet did not yield crystals. Modifications of the construct and the buffer remained unsuccessful and therefore the construct sequence was analysed using the Surface-Entropy-Reduction prediction (SERp) server [31] for patches of surface residues, which, once mutated, could potentially promote local stability, and the three highest scoring mutants were tested for expression and crystallisation. One of them, the double mutant EE709AA, dubbed construct HMA6^N^-AA, led, together with a change of the buffer, to an initial crystal hit. Yet the crystals proved difficult to optimize, grew in clusters, required microseeding in order to reproduce the hit and only diffracted to 3.2 Å, with six molecules in the AU in space group *P*3_2_21. A reduction of the protein concentration to 4 mg.mL^-1^ occasionally led to crystals with a different, more wedge like shape and a different space group (*P*22_1_2_1)_, not showing any more the inter-grown clusters seen initially. These crystals diffracted up to 1.5 Å resolution and contained only two molecules in the AU. For both proteins, two molecules are present in the AU, yet they are monomeric in solution as found by size exclusion chromatography. The HMA6 and HMA8 nucleotide binding and phosphorylation domains (N/P domain, HMA6^N/P^, residues 576–853 and HMA8^N/P^, residues 526–806) were expressed and purified, yet in both cases a fraction of the protein was found to be *C*-terminally truncated. While the full N/P domains were dimeric in solution the truncated fragments were monomeric. Upon removal of the truncated fraction, the full-length constructs were stable, yet did not crystallize.

### Structure of HMA6 and HMA8 in comparison to others

**Fig 1:**
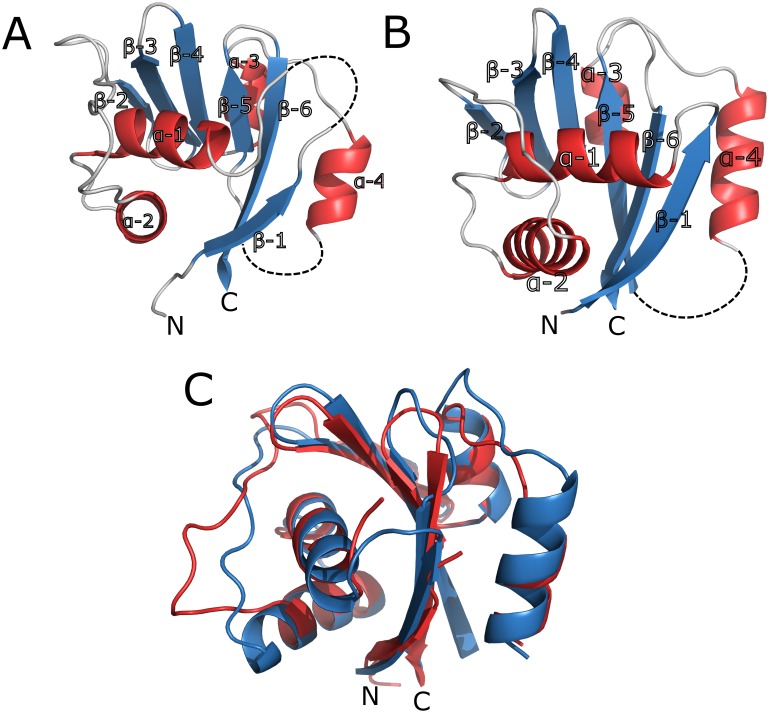
3D structures of HMA6 and HMA8 N-domains. **A** The structure of HMA6^N^-AA with β-strands shown in blue and α-helices in red. Missing loops are outlined by dashed lines. **B** The structure of HMA8^N^; colouring is as in A. **C** Superimposition of the two structures with HMA6^N^-AA in red and HMA8^N^ in blue.

**Fig 2:**
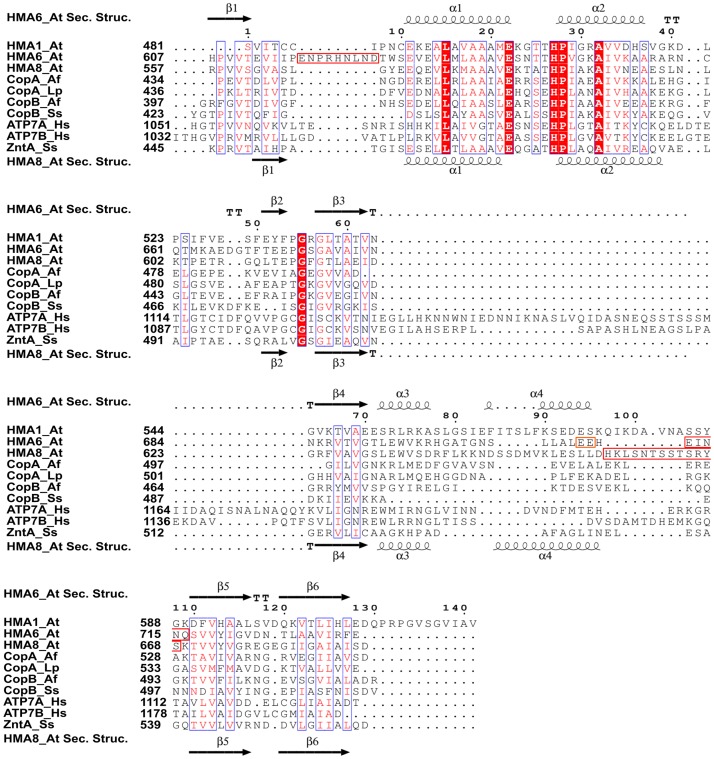
Sequence alignment of P_IB_-type ATPase N-domains. Alignment of the region covering the N-domains of HMA1, HMA6 and HMA8 from *A*. *thaliana*, CopA and CopB from *A*. *fulgidus*, CopA from *L*. *pneumophila*, CopB from *S*. *solfataricus*, the human Menkes (ATP7A) and Wilson proteins (ATP7B) and ZntA from *S*. *sonnei*. Disordered regions in HMA6 and HMA8 are marked by a red rectangle. The mutated residues in HMA6 are marked in orange. The secondary structures of HMA6 and HMA8 are indicated above and below the alignment, respectively. Multiple sequence alignment was done using MUSCLE (http://www.ebi.ac.uk/Tools/msa/muscle) and visualized using ESPript 3.0 [35].

**Fig 3:**
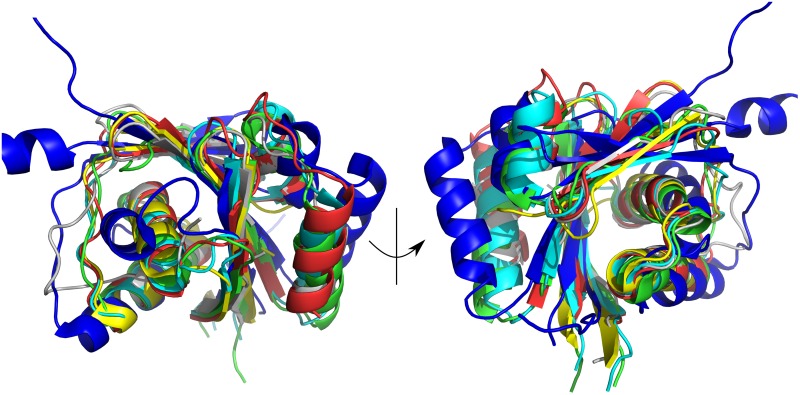
Comparison of P_IB_-type ATPase N-domains of structural homologs. Superimposition of HMA6^N^-AA (gray), HMA8^N^ (red), LpCopA (3RFU, green), AfCopA (2B8E, cyan) and AfCopB (3SKX, orange), SsCopB (2IYE, yellow) and the Wilson disease protein (2ARF, blue).

The overall structure of HMA6^N^-AA and HMA8^N^ (Fig 1A and 1B, respectively) exhibits a twisted, antiparallel β-sheet made of six strands being flanked by two pairs of short α-helices, one on the concave (α1 and α2) and one on the convex (α3 and α4) side of the sheet (Fig 1C). The overall fold is similar to those found in other P-type ATPases [17,32] (Tables 3 and 4). While the central β-sheet is highly conserved within the subfamily, the main differences, as in the Menkes and Wilson proteins [33,34], arise in the loop length and the number of helices (Figs 2 and 3). In HMA6^N^-AA two loops are disordered—L1 between β1 and α1 (res. 617–625) and L5 between α4 and β5 (res. 712–716, with α4 partly and completely disordered in chain A and B, respectively), while in HMA8^N^ only one loop is flexible (res. 656–668). The first missing loop in HMA6 corresponds to an insertion not present in most other P_IB_-type ATPases (Fig 2). The position of the second missing loop is identical in both structures, differing only in the length, as in HMA8 an insertion is present, not typically found in the subfamily (Fig 2). The position of helix α4, which precedes the loop and is also partially disordered, relative to a helix in the P-domain is indicative of the conformation, open or closed, of the two domains. While not specific contacts are present in structures where the N- and P-domain are present, the P-domain could aid in stabilizing this disordered region. Furthermore, the insertion found in HMA8 could interact tighter with the P-domain and report on the N-domain inclination. In the two HMA structures helix, α4 is more tightly packed against the β-sheet than in other members of the subfamily, which doesn’t seem to be caused by the absence of the P-domain, as other isolated N-domains show a less tight packing [33,34]. Within the AU, both structures are highly similar with an rmsd of 0.43 Å and 0.33 Å for HMA6^N^-AA and HMA8^N^, respectively. When comparing the two proteins they also show a high similarity with an rmsd of about 1 Å (Fig 1).

**Table 3:** A matrix of sequence identity values among the P_IB_-type ATPase N-domains compared to HMA (1, 6 and 8) N-domains.

	HMA1^N^	HMA6^N^	HMA8^N^	LpCopA	AfCopA	AfCopB	SsCopB	ATP7A	ATP7B
HMA1N	-	29	23	22.6	20.3	25.4	18.3	15.6	18.9
HMA6N	29	-	34.2	28.5	27.7	28.7	26.1	27.5	23.3
HMA8N	23	34.2	-	23.1	31.3	32.8	31.2	23.3	24.2
LpCopA	22.6	28.5	23.1	-	32.7	34.2	28.6	26.5	24.8
AfCopA	20.3	27.7	31.3	32.7	-	40.5	35.2	31.3	29.6
AfCopB	25.4	28.7	32.8	34.2	40.5	-	34.7	26.3	23.9
SsCopB	18.3	26.1	31.2	28.6	35.2	34.7	-	26	28.4
ATP7A	15.6	27.5	23.3	26.5	31.3	26.3	26	-	54
ATP7B	18.9	23.3	24.2	24.8	29.6	23.9	28.4	54	-

**Table 4:** A matrix of RMSD values (in Å) among the N-domains of structural homologs of HMA6^N^ and HMA8^N^ compared in Fig 3 using the CA positions.

	HMA6^N^ 5LBD	HMA8^N^ 5LBK	LpCopA 3RFU	AfCopA 2B8E	AfCopB 3SKX	SsCopB 2IYE	ATP7B 2ARF
HMA6^N^ 5LBD	-	0.73	1.04	0.77	1.35	1.14	4.02
HMA8^N^ 5LBK	0.73	-	1.19	2.9	0.92	0.87	5.86
LpCopA 3RFU	1.04	1.19	-	1.26	1.32	1.22	3.6
AfCopA 2B8E	0.77	2.9	1.26	-	1.51	1.95	3.44
AfCopB 3SKX	1.35	0.92	1.32	1.51	-	1.05	4.39
SsCopB 2IYE	1.14	0.87	1.22	1.95	1.05	-	4.01
ATP7B 2ARF	4.02	5.86	3.6	3.44	4.39	4.01	-

### Effects of surface mutants

**Fig 4:**
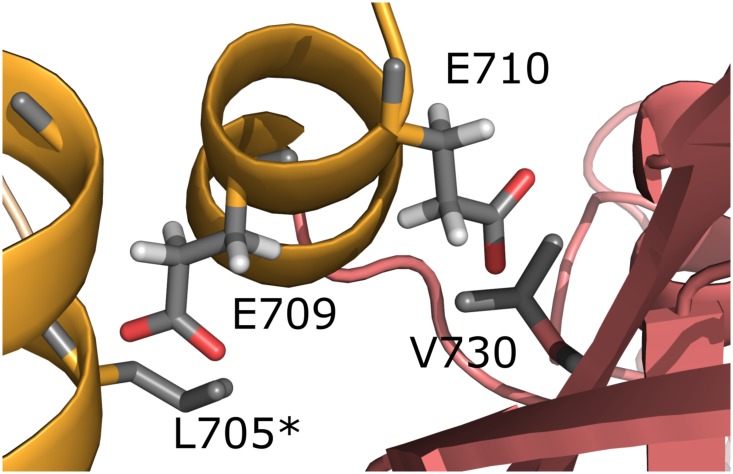
Effect of the double alanine mutant in HMA6^N^. Interface of helix 4 from chain A and the symmetry related chain A* with glutamate modelled at position 709 and 710, matching the WT sequence instead of the two alanine, as found in the structure. Helix 4 is shown in orange in the symmetry related side chain marked with an asterisk.

The effect of the double alanine mutant in HMA6, proposed by the SERp server, seems to have two aspects. Glu709A, in the partially disordered helix α4, points to the solvent and the glutamate of the wild type sequence, when modelled in the structure, clashes with the side chain of Leu705 of the symmetry related chain A* (Fig 4). Likewise, the same clash of Glu709 of chain A* with Leu705A would occur. On the other hand, the side chain of Glu710 points towards the centre of the domain and would clash with Val730 of β6. If not mutated glutamate 709 and 710 would be 1.2–1.5Å from the next side chain, with the clash preventing the crystal formation. Several crystal contacts are present between the chains in two axis, while these mutations allowed for a crystal contact in the third axis, between α4 and α4'. The same region in chain B, including helix α4 (704–716), is disordered, potentially caused by the lack, unlike for chain A, of a stabilizing interaction with a symmetry related chain.

### ATP binding properties

ATP binding to the isolated N-domain of HMA6 and HMA8 was assessed by ITC in different experimental conditions (various proteins and ATP concentrations, presence or absence of magnesium). None of them showed a signal of interaction between HMA6^N^ or HMA8^N^ and ATP. In parallel, several attempts to obtain the structure of the HMA N-domains in complex with ATP were made by soaking or co-crystallization on HMA8^N^. They led to diffracting crystals but no density of the nucleotide could be observed. An accurate review of the literature shows that nucleotide binding studies of isolated N-domains of P-type ATPases are scarce. A few data have been reported such as on the rat Na^+^K^+^-ATPase from rat [36], the rabbit skeletal muscle Ca^2+^-ATPase SERCA1a [37] and the K^+^-ATPase KdpB from *E*. *coli* [38]. Apparent ATP affinities measured on these N-domains are low (Kd of 5–25 mM for the Na^+^K^+^-ATPase; 0.7–2.4 mM for SERCA1a; 1.4 mM for KdpB), up to 10 times lower than the apparent affinity determined with the full-length proteins. For a given protein the range of Kd values is sometimes large since measured by different methods (Chemical shift, ITC, intrinsic fluorescence). These values do not depend on the presence of magnesium except for the Na^+^K^+^-ATPase N-domain that unexpectedly exhibits a lower affinity for ATP in presence of magnesium.

Similar studies have also been carried out on the highly homologous human Cu^+^-ATPases ATP7B [34,39–41] and ATP7A [33]. The N-domains of these two proteins were shown to display similar apparent Kd for ATP ranging from 10 to 110 μM. The data available on AfCopA [17,42–44] and ssCopB [32] were obtained on the [N + P]-domains and therefore cannot be strictly compared with the present work in terms of ATP binding.

A striking difference between the N-domains of the human and plant Cu^+^-ATPases is the presence of a large unfolded region found between β3 and β4 only found in ATP7A and ATP7B (Fig 2). Such a region is also missing in the N-domains of prokaryotic Cu^+^-ATPases such as AfCopA for which no ATP binding has been reported. This additional region found in the N-domains of mammalian Cu^+^-ATPases even though not directly involved in nucleotide binding could give some flexibility to the isolated domain allowing a measurable binding of ATP. Indeed, it has been shown in ATP7A that decrease in size of the adenine ring binding cavity caused by the motion of both α1- α2 and β2-β3 loops, allows a tight packing around the ATP molecule [33]. So, even faint, a conformation change of the isolated N-domain is required for ATP binding in ATP7A (and probably ATP7B).

A detailed analysis of the nucleotide binding site among the Cu^+^-ATPases is presented below.

### Nucleotide binding site analysis

**Fig 5:**
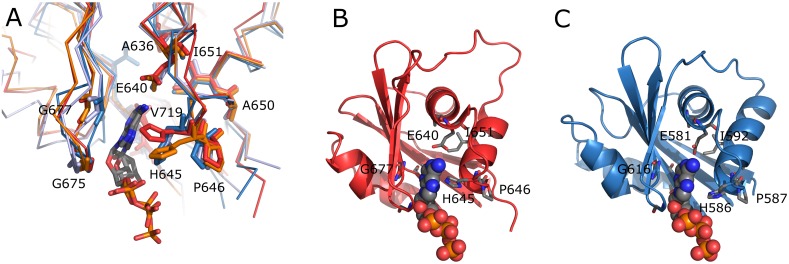
**A Superimposition of the ATP binding site of HMA6**^**N**^**-AA (red), HMA8**^**N**^ **(orange), SsCopB (blue, 2YJ4) and AfCopA (light-blue, 3A1D)**. Conserved residues are shown as sticks, coloured as the corresponding chain and numbered according to their position in HMA6. ATP and ADP of SsCopB and AfCopA are shown as sticks with carbon atoms show in grey, nitrogen atoms in blue, oxygen atoms in red and phosphate atoms in orange. **B** Structure of HMA6^N^-AA and **C** HMA8^N^, each with the ATP of the SsCopB structure modelled. For the positioning of the nucleotide the N-domains of HMA6^N^-AA, HMA8^N^ and SsCopB (2YJ4) were superimposed and as the conserved residues are spatially in close proximity a model of HMA6^N^-AA and HMA8^N^ together with the ATP of SsCopB was generated. Conserved residues important for the binding are shown in sticks and the ATP as spheres. Colouring as in A.

The HMA N-domains structures were compared to the [N + P] domains structures of the two prokaryotic Cu^+^-ATPases AfCopA (pdb 3A1D) and SsCopB (pdb 2YJ4) that were solved in the presence of ATP or ADP. No major structural changes are expected upon the binding of ATP. A superimposition shows that the nucleotide binding site and the position of conserved residues is basically identical between the structures (Fig 5A) as well as a good fit of the binding pocket to the nucleotide. The three conserved glycine residues G675, G677 and G690 (HMA6 numbering) lining one side of the binding pocket and the glutamate residue E640 (HMA6 numbering), normally in contact with the N1 and N6 atoms of the nucleotide, are occupying positions ready for nucleotide binding. In contrast, histidine H645 (HMA6 numbering), part of the HP motif and found elsewhere in contact with the α– and β-phosphate [17], is pointing away from the binding site and would have to adopt a different position for proper interaction with the phosphate chain (Fig 5B and 5C). Of all available structures of the P_1B_ subfamily, a part of the histidine residues is already in such a position, while the remaining adopt an orientation similar to the one observed in the HMA structures. Therefore, it seems that the proper orientation of the histidine is not a consequence of the nucleotide binding but rather a prerequisite, possibly aided by the P-domain, providing a conceivable explanation for our failure to observe ATP binding to the isolated HMA domains. In summary the modelled position of ATP in the N-domain (Fig 5B and 5C) most likely provides an accurate description, while changes in the side chain orientation of the histidine H645 (HMA6 numbering) are needed for proper interaction.

An interesting observation in the case of HMA6 is the presence of glutamate 672, not commonly found at this position, pointing to the nucleotide binding cavity and guiding the position of the conserved H645. While missing in HMA8, this interaction probably contributes to the inability of the isolated domain to bind ATP. Of interest is also a second salt bridge in the vicinity of the nucleotide binding domain, E673-R697 in HMA6 and E612-R637 in HMA8, linking β2 to α3. A salt bridge is also observed in LpCopA (pdb 3RFU), however the charges are inverted (K493-E514), while in the Menkes protein, only van der Waals contacts are present (pdb 2KMX). This bridge sits either next to, or interacts via, the backbone with the highly conserved glycine in β3 (677 in HMA6, 616 in HMA8 or 494 in LpCopA), part of a highly flat region of the β-sheet in contact with the adenine ring, and probably aides in positioning of this glycine. While the loop between β2 and β3 is not changing its position upon nucleotide binding in these structures, it is moving in the case of SsCopB (pdb 2IYE), which lacks helices 3 and 4 and is not stabilized by the mentioned salt-bridge. The salt bridge could therefore control the position of the two strands β2-β3 relative to the cavity. Interestingly, the charge distributions at the surface delineating the cavity differ from the known N-domain structures (S1 Fig). Electrostatic properties of this surface, might not only guide the entrance of the nucleotide to the cavity and the interactions with the P-domain, but also influence the kinetic properties of these pumps. Marked differences between ATP/ADP ratios in the cytosol and the chloroplast stroma [45], as well as between the pH of these two subcellular compartments (increase of the stromal alkalization on illumination), might also explain differences in electrostatic properties of this surface (HMA8 requires the efficient use of ATP at a low ATP/ADP ratio found in the stroma).

Although too few data are yet available on Cu^+^-ATPases to draw a general conclusion on their ATP binding mechanism, it sounds realistic to correlate the affinity of ATP to the ability of the isolated N-domain to accommodate efficiently the nucleotide and therefore to adapt its conformation. In that sense, our study reveals that the N-domains of AtHMA6 and AtHMA8 exhibit significant differences compared to those of ATP7A and ATP7B, the only Cu^+^-ATPases for which ATP binding was measured on the isolated N-domains. It also suggests that ATP binding mechanism of AtHMA6 and AtHMA8 (and by extension to prokaryotic Cu+-ATPases) might resemble more that of KdpB.

## Supporting Information

S1 Fig Electrostatic potential surfaces of P1B-type ATPases. Red denotes negative and blue positive charges, respectively. All structures are limited to the N-domains and oriented the same way, with the nucleotide binding site in the centre and the nucleotide, if present, shown as sticks. For each structure the pdb code is given. In summary the first structures of plant P1B-type ATPase N-domains show their high similarity to their bacterial counterparts, with the binding amino acids in place for interaction with ATP. The central histidine of the HP motif needs to be reoriented in order to interact with the adenine ring, providing a possible clue why these isolated domains show a very weak affinity for ATP. In addition, a so far not noted salt bridge linking the central β-sheet to α3 stabilizes the glycine. Finally, modifications of the surface helped in obtaining crystals of the HMA6 N-domain that were otherwise unattainable.
(TIF)
