# Supplementary figures and images for: Structural Insights into the Nucleotide-Binding Domains of the P1B-type ATPases HMA6 and HMA8 from Arabidopsis thaliana
Source: PLoS One. 2016 Nov 1;11(11):e0165666. doi: 10.1371/journal.pone.0165666 (PMC5089723; doi:10.1371/journal.pone.0165666)

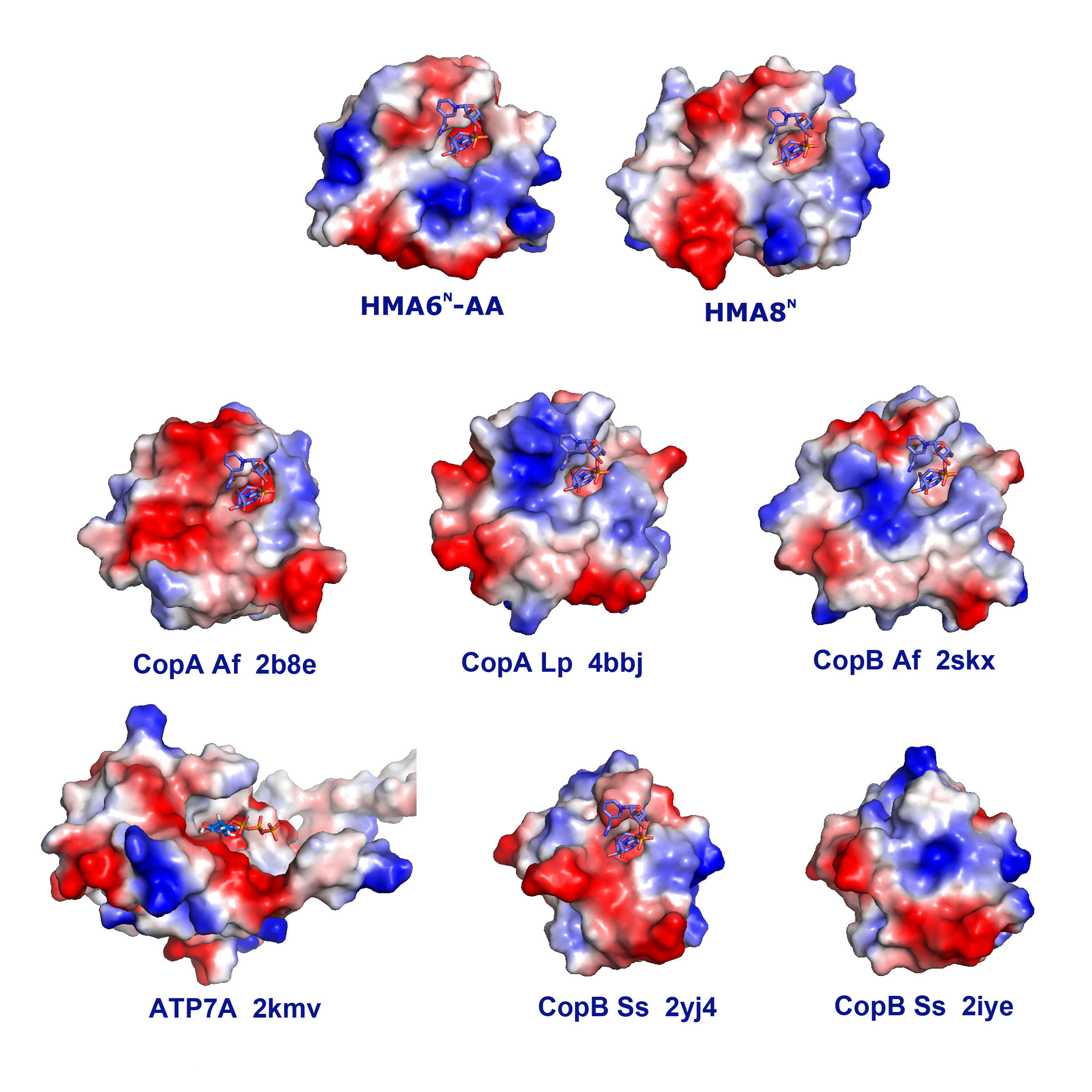

Supplement: S1 Fig — Red denotes negative and blue positive charges, respectively. All structures are limited to the N-domains and oriented the same way, with the nucleotide binding site in the centre and the nucleotide, if present, shown as sticks. For each structure the pdb code is given. In summary the first structures of plant P1B-type ATPase N-domains show their high similarity to their bacterial counterparts, with the binding amino acids in place for interaction with ATP. The central histidine of the HP motif needs to be reoriented in order to interact with the adenine ring, providing a possible clue why these isolated domains show a very weak affinity for ATP. In addition, a so far not noted salt bridge linking the central β-sheet to α3 stabilizes the glycine. Finally, modifications of the surface helped in obtaining crystals of the HMA6 N-domain that were otherwise unattainable. (TIF) [file pone.0165666.s001.tif]
